# Editorial: Adaptation of halophilic/halotolerant microorganisms and their applications, volume II

**DOI:** 10.3389/fmicb.2026.1780220

**Published:** 2026-01-29

**Authors:** Rosa María Martínez-Espinosa, Sumit Kumar, Shiladitya DasSarma

**Affiliations:** 1 Department of Biochemistry and Molecular Biology and Edaphology and Agricultural Chemistry, Faculty of Sciences, University of Alicante, Alicante, Spain; 2 Multidisciplinary Institute for Environmental Studies “Ramón Margalef” (IMEM), University of Alicante, Alicante, Spain; 3 Amity Institute of Biotechnology, Amity University Uttar Pradesh, Noida, India; 4 School of Medicine, University of Maryland Baltimore, Baltimore, MD, United States

**Keywords:** halophiles in agriculture, halophilic microorganisms, halotolerant microorganisms, high salt adaptation, saline environment, saline soils, salt stress tolerance, salt-tolerant bacteria

Halophilic and halotolerant microorganisms have uniquely adapted to thrive in high-salt environments, such as saline soils, deserts, salt lakes, and certain food products. Their ability to adapt to extreme conditions has garnered significant interest in various fields, including agriculture, medicine, and biotechnology. Recent advancements in the isolation and taxonomic characterization of these microorganisms, along with studies on their metabolic capabilities and the compounds they produce, have greatly expanded our understanding of their diversity and resilience. Moreover, exploring the microbial diversity of Earth's extreme environments contributes to our understanding of how life could be possible on other planets. Related to this topic, Runzheimer et al. analyzed microbial diversity in Zechstein salt deposits located in Germany. Their findings reveal that the most abundant microbial communities include sulfate-reducing bacteria, haloarchaea, and as-yet-uncultivated microorganisms such as *Nanohaloarchaeota* and *Patescibacteria*, all of which are of interest for biotechnological applications and astrobiological studies.

Using omics approaches, the microbial diversity of saline and freshwater lakes in the Bolivian Altiplano and the molecular adaptation to athalassohaline conditions in Lake Barkol (China) were explored. Hentschke et al. chose the Bolivian Altiplano because it presents polyextreme conditions. The aim of their study was to describe cyanobacterial mats and their associated microbiomes, thus being the first comprehensive metabarcoding analysis of cyanobacterial mats from Bolivia. The main findings confirm that the microbiome associated with these mats is dominated by *Alphaproteobacteria, Bacteroidia, Gammaproteobacteria, Clostridia, Cyanophyceae*, and *Campylobacteria*, all of them showing interesting potential applications in biotechnology and playing relevant roles in biogeochemical cycling under polyextreme conditions. Conversely, studies carried out at Lake Barkol by Xamxidin et al. resulted in the assembling of 309 genomes, 279 of which correspond to bacterial species and 30 to archaeal species. In addition to taxonomic identification, the metabolic reconstruction revealed the presence of diverse carbon fixation pathways, nitrogen fixation, sulfur oxidation/reduction, and denitrification. The authors' main conclusion is that salinity plays a relevant role in structuring microbial assemblages and metabolic pathways. This pattern was also reported in the metagenomics-based research conducted by Li et al., who monitored microbial metabolic functions in salinized soils and their response to salinity in arid regions of Xinjiang (China). The main results confirm that salinity in arid regions exerts significant control over the expression of genes related to nutrient cycling so that it inhibits multiple metabolic functions but promotes the function of carbon fixation, denitrification, ANRA, and organic phosphorus dissolution.

As the global population continues to grow, the increasing salinization of soils poses a serious threat to food security. Thus, developing strategies to overcome salinity-induced agricultural problems is of vital importance. Halophilic and halotolerant microorganisms offer promising solutions by enhancing plant growth in saline environments. These microorganisms produce plant-active compounds (such as indole-3-acetic acid, hydrogen cyanide, 1-aminocyclopropane-1-carboxylate–deaminase, and exopolysaccharides), fix atmospheric nitrogen, and solubilize plant nutrients, such as phosphate. In this context, Tiwari et al. conducted a study to identify and evaluate native microbial communities from salt-affected regions (in eastern Uttar Pradesh, India) to boost black gram (*Vigna mungo*) resilience against salinity, while improving plant growth. Among the bacterial strains isolated, co-inoculation with *Bradyrhizobium yuanmingense* PR3 and *Paenibacillus* sp. SPR11 results in a significant enhancement of seed germination and root and shoot growth, thus evidencing promising potential to alleviate salt stress and enhance plant growth.

Halophilic and halotolerant microorganisms also have potential applications in biotechnology. Their enzymes, including lipases, proteases, esterases, nucleases, and hydrolases, demonstrate activity across a broad range of pH, salt, and temperature conditions. This versatility makes them ideal for use in the food and pharmaceutical industries, in biofuel production, and in the bioremediation of wastewater and contaminated soils. To search for extremophilic enzymes for biotechnological applications, Hoepfner et al. utilized shotgun metagenomics and functional annotation to explore enzymatic diversity across an 8-meter depth gradient in the Uyuni Salt Flat (Bolivia). The results show that while amylases predominate at the surface, lipases and peroxidases are more abundant in intermediate depths. Deeper layers are rich in peptidases and peroxidases. This study not only demonstrates functional stratification but also offers new options for the biotechnological use of extreme enzymes. In connection with biotechnological applications of extreme enzymes, Foronda et al. made efforts to study the production of lipases by *Bacillus safensis* VC-6, which was isolated from the volcanic region of Copahue (Chile). The findings demonstrate that this strain exhibits greater potential for lipase production than other *Bacillus* species, and its lipases exhibit higher thermostability and halotolerant profiles. Considering the applications of these enzymes, this study was complemented with the optimization of growth conditions and the identification of critical genes related to lipase production to further enhance their production at a large scale. In the context of bioremediation, Wang et al. optimized an adaptive laboratory evolution process for phenol degradation in the presence of high salt concentrations using a co-culture of *Halomonas mongoliensis* and *Dunaliella salina*. This study demonstrated that the co-culture system can completely degrade 500 mg L^−1^ of phenol within 8 days, outperforming the original *D. salina* co-culture system.

Finally, the antioxidants, antimicrobial molecules, and small peptides showing different biological activities produced by these microorganisms have applications in biomedicine and the food industry, while their compatible solutes are valuable in the cosmetic and pharmaceutical sectors. These solutes not only mitigate desiccation damage and protect proteins under extreme conditions (such as high or low temperatures and oxidative stress), but also regulate gene expression in cells (such as heat shock proteins in keratinocytes). Consistent with these applications, Karthik et al. isolated *Boudabousia marimammalium* from mangrove soil located in the Mangalore region of Dakshina Kannada (India). This microorganism has the capacity to produce bioactive peptides with dual antimicrobial (against some of the most common bacterial genera that cause human diseases) and anticancer properties (tested against PC3 cells, which are representative of prostate cancer in humans).

In summary, advances in knowledge about the molecular adaptations of halotolerant and halophilic microorganisms not only allow us to better understand ecosystem dynamics but also highlight their enormous potential for developing processes that allow us to minimize the impact of environmental pollution and climate change, without forgetting the possibility of designing and developing processes and formulations that integrate their molecules. A clear example of the enormous potential offered by these microorganisms is the significant number of research articles published each year on this topic, due to the social and scientific impact of the results obtained and the investments made worldwide by different agencies to support research in this field.

